# Uncovering the DNA methylation landscape in key regulatory regions within the *FADS* cluster

**DOI:** 10.1371/journal.pone.0180903

**Published:** 2017-09-28

**Authors:** Elaheh Rahbar, Hannah C. Ainsworth, Timothy D. Howard, Gregory A. Hawkins, Ingo Ruczinski, Rasika Mathias, Michael C. Seeds, Susan Sergeant, James E. Hixson, David M. Herrington, Carl D. Langefeld, Floyd H. Chilton

**Affiliations:** 1 Department of Biomedical Engineering, Wake Forest School of Medicine, Winston-Salem, NC, United States of America; 2 Department of Biostatistical Sciences, Division of Public Health Sciences, Wake Forest School of Medicine, Winston-Salem, NC, United States of America; 3 Center for Genomics and Personalized Medicine Research, Wake Forest School of Medicine, Winston-Salem, NC, United States of America; 4 Department of Biostatistics, Johns Hopkins Bloomberg School of Public Health, Baltimore, MD, United States of America; 5 Division of Allergy and Clinical Immunology Department of Medicine, The Johns Hopkins University, Baltimore, MD, United States of America; 6 Department of Internal Medicine, Section on Molecular Medicine, Wake Forest School of Medicine, Winston-Salem, NC, United States of America; 7 Department of Biochemistry, Wake Forest School of Medicine, Winston-Salem, NC, United States of America; 8 Department of Epidemiology, Human Genetics and Environmental Sciences, Human Genetics Center, School of Public Health, University of Texas Health Science Center at Houston, Houston, TX, United States of America; 9 Department of Internal Medicine, Division of Cardiology, Wake Forest School of Medicine, Winston-Salem, NC, United States of America; 10 Department of Physiology and Pharmacology, Wake Forest School of Medicine, Winston-Salem, NC, United States of America; National Cancer Institute, UNITED STATES

## Abstract

Genetic variants near and within the fatty acid desaturase (*FADS*) cluster are associated with polyunsaturated fatty acid (PUFA) biosynthesis, levels of several disease biomarkers and risk of human disease. However, determining the functional mechanisms by which these genetic variants impact PUFA levels remains a challenge. Utilizing an Illumina 450K array, we previously reported strong allele-specific methylation (ASM) associations (p = 2.69×10^−29^) between a single nucleotide polymorphism (SNP) rs174537 and DNA methylation of CpG sites located in the putative enhancer region between *FADS1* and *FADS2*, in human liver tissue. However, this array only featured 20 CpG sites within this 12kb region. To better understand the methylation landscape within this region, we conducted bisulfite sequencing of the region between *FADS1* and *FADS2*. Liver tissues from 50 male subjects (27 European Americans, 23 African Americans) were obtained from the Pathobiological Determinants of Atherosclerosis in Youth (PDAY) study, and used to ascertain the genotype at rs174537 and methylation status across the region of interest. Associations between rs174537 genotype and methylation status of 136 CpG sites were determined. Age-adjusted linear regressions were used to assess ASM associations with rs174537 genotype. The majority of CpG sites (117 out of 136, 86%) exhibited high levels of methylation with the greatest variability observed at three key regulatory regions–the promoter regions for *FADS1* and *FADS2* and a putative enhancer site between the two genes. Eight CpG sites within the putative enhancer region displayed significant (FDR p <0.05) ASM associations with rs174537. These data support the concept that both genetic and epigenetic factors regulate PUFA biosynthesis, and raise fundamental questions as to how genetic variants such as rs174537 impact DNA methylation in distant regulatory regions, and ultimately the capacity of tissues to synthesize PUFAs.

## Introduction

**Fig 1:**
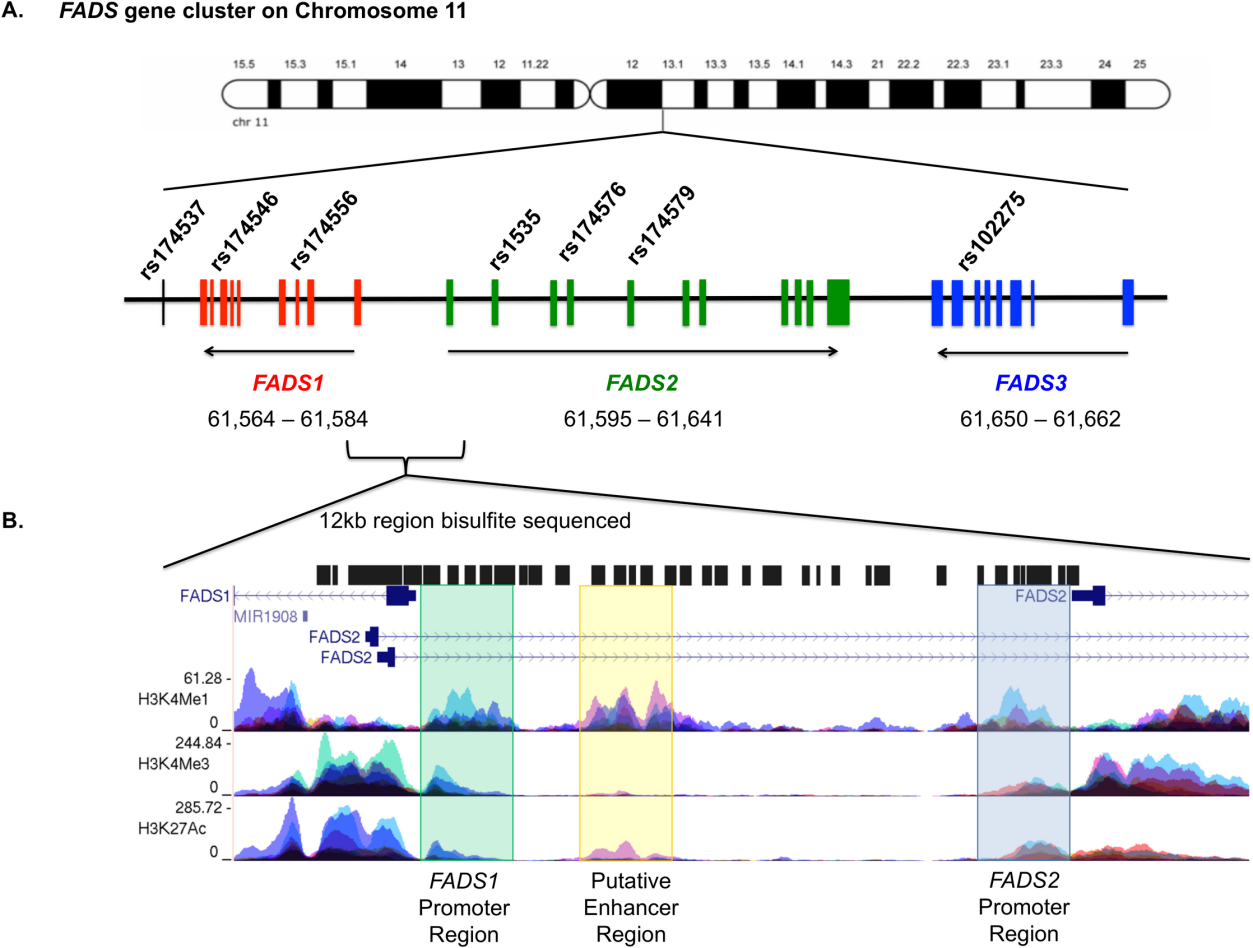
Illustration of *FADS* gene cluster and sequenced region of interest. (A) Illustration of *FADS* gene cluster on chromosome 11 with seven key SNPs in the *FADS* cluster marked. (B) Illustration of region between *FADS1* and *FADS2* that was bisulfite sequenced (61,584,720–61,595,166) with three key regulatory regions highlighted, namely: *FADS1* promoter region (green), putative Enhancer region (yellow) and *FADS2* promoter region (blue).

Polyunsaturated fatty acids (PUFAs) are vital for normal growth and development, serving as key structural components of biological membranes and modulating critical signal transduction events. In particular, long-chain PUFAs (LC-PUFAs) are precursors of bioactive metabolites, which have been implicated in several human diseases including cardiovascular disease, cancer, and inflammation [1–4]. Historically, the metabolic conversion and endogenous synthesis of LC-PUFAs from dietary PUFAs has been considered to be slow and relatively uniform in humans. Studies over the past decade [5–27] have demonstrated that genetic and epigenetic variations near and throughout the fatty acid desaturase (*FADS)* gene cluster (Fig 1) account for large variations in circulating and cellular LC-PUFA levels. Several common polymorphisms within the *FADS* gene cluster, including the single nucleotide polymorphism (SNP) rs174537, have been shown to be highly associated with circulating and tissue PUFA levels and PUFA product-to-precursor ratios [9–11, 28, 29]. In light of these studies, the conventional paradigm of slow, inefficient and uniform LC-PUFA biosynthesis in humans has been challenged.

Genetic variants in the *FADS* gene cluster have also shown strong associations with intermediate biomarkers of disease, such as total cholesterol, low-density lipoprotein (LDL), high-density lipoprotein (HDL), triglycerides, phospholipids, C-reactive protein, and pro-inflammatory eicosanoids [30, 31] and additionally, several complex diseases, including coronary artery disease [17, 25, 32], metabolic syndrome [11], allergic rhinitis, atopic eczema [26], and attention deficit/hyperactivity disorder [33]. Most notable is rs174537, located approximately 15kb downstream of *FADS1*, which displays the strongest association (p<10^−40^) with blood and tissue levels of arachidonic acid (ARA; C20:4n-6) and the metabolic conversion capacity, represented by product to precursor ratios (e.g. ARA/DGLA) [11, 29, 34]. In addition, there are marked allele frequency differences of rs174537 between racial/ethnic groups, with African populations having a derived haplotype that is associated with higher ARA levels and more efficient LC-PUFA biosynthesis [11, 35, 36]. Together, these studies reveal the importance of genomic factors on PUFA biosynthesis and chronic inflammatory diseases in racially diverse populations.

Despite consistent associations between SNPs near and in the *FADS* cluster and LC-PUFA levels and related functional phenotypes, much remains unknown regarding the molecular mechanism of these genomic variants on PUFA metabolism in critical tissues and organs. A few studies indicate that genetic variants such as rs174537, and SNPs in high linkage disequilibrium (LD) with rs174537, may alter LC-PUFA levels via changes in *FADS* gene expression levels. This could occur in several ways, including altering the regulatory landscape (e.g., promoter or enhancer) of a gene, alternative RNA splicing, transcript degradation, or transcription of non-coding RNA. Additionally, epigenetic modifications such as DNA methylation may influence *FADS* gene expression levels. We recently performed a genome-wide, allele-specific methylation (ASM) analysis with rs174537 in human liver tissue to test the hypothesis that rs174537 (or variants in high LD) interacts with key regulatory regions within the *FADS* cluster and be associated with DNA methylation levels in a mechanistically important way [34]. Utilizing the Illumina HumanMethylation450 BeadChip, only one region of the genome (within the 12kb region located between *FADS1* and *FADS2*), on chromosome 11q12.2, showed an ASM association with rs174537 in both African Americans and European Americans. This strong association was observed with methylation site cg27386326 (i.e., 61587979 in this manuscript) (p<10^−20^), which is located ~3.5kb from the *FADS1* and ~7.8kb from the *FADS2* transcription initiation sites, in a region with a putative enhancer signature, near transcription factor binding sites for c-Fos, STAT3 and MafK. The methylation proportion differed by 0.40 between homozygotes at rs174537 (0.44, GG genotype; 0.84, TT genotype) and was highly associated with LC-PUFA biosynthesis.

A limitation of the aforementioned study [34] was that the Illumina HumanMethylation450 BeadChip (485,577 CpG sites) only featured a small number of probes (N = 20) within the potentially important 12kb regulatory region between *FADS1* and *FADS2*. The 12kb region between *FADS1* and *FADS2*, which are transcribed in opposite directions, contains three key regions of interest: (i) the *FADS1* promoter region (approximately chr11: 61,584,650–61,586,300), (ii) a putative enhancer region (approximately chr11: 61,587,300–61,589,000), and (iii) the *FADS2* promoter region (approximately chr11: 61,594,300–61,595,600) (Fig 1). To comprehensively examine the potential associations between the rs174537 genotypes and the methylation of all CpG sites within this regulatory region, we conducted bisulfite sequencing of the 12kb region between *FADS1* and *FADS2*. Our goal was to characterize the DNA methylation landscape of this regulatory region and identify any new ASM associations between rs174537 and CpG sites within this region.

## Materials and methods

### Study samples

DNA and liver tissue from 50 individuals (27 European American and 23 African American) were obtained from the Pathobiological Determinants of Atherosclerosis in Youth (PDAY) study [37]. PDAY was an autopsy study designed to enroll men and women between 15–34 years or age who died of non-cardiovascular disease related causes (e.g., traumatic injuries). Autopsies were performed within 72 hours of death, and livers were frozen at -80°C. DNA was then isolated from these tissue samples, as previously described [37]. Since the original PDAY study was geared towards atherosclerosis, levels of HDL and non-HDL, thiocyanate and glycohemoglobin were quantified. Age, body mass index (BMI) and whether the patient was hypertensive were also documented. Since all study subjects were deceased at the time of study, use of these liver tissue specimens is not considered human subjects research.

### Genotyping and DNA methylation analysis

Liver tissue samples were originally genotyped at rs174537 and evaluated for DNA methylation using the Illumina HumanMethylation450 BeadChip, as previously described [34]. A portion of the 12kb region between *FADS1* and *FADS2* (Fig 1B) was bisulfite sequenced by Zymo Research, Inc. After evaluation of repeat sequences and allowing for PCR failures, ~10.5kb out of the 12kb region could be sequenced, and 136 CpG sites were reliably quantitated. Methylation levels ≤5% were excluded across all samples, based on the assay’s limit of detection and sensitivity. The name of each CpG is based on its genomic position on chromosome 11 (0-based format), based on genome build GRCh37/hg19. All methylation data from this study cohort has been provided as a supplemental file (S1 File).

### Statistical analysis

ASM analysis was conducted to identify CpG sites most associated with the genotype at rs174537. By ethnicity, each of the 136 CpG sites was evaluated for an association with rs174537 using a linear regression model adjusted for age. Genotypes for rs174537 were coded for a dominant genetic model, relative to the T-allele. CpG site associations in the two cohorts were then analyzed in a trans-ethnic meta-analysis. The meta-analysis was computed using METAL, which implements a weighted inverse normal method (weighted by sample size) [38]. For each analysis (ethnic-specific and meta-analysis), the Bonjamini-Hochberg FDR (BH-FDR) p-values were calculated and reported along with the raw p-values. Significance was set at the 0.05 BH-FDR level. All statistical analyses were conducted using commercially available software (STATA and SAS) and open source statistical software (R).

## Results

DNA methylation throughout the sequenced region was characterized, and included 136 CpG sites that spanned the three key regulatory regions: (i) the *FADS1* promoter region, (ii) the putative enhancer region and (iii) the *FADS2* promoter region (Fig 1). DNA methylation analyses were successfully performed on all 50 individuals (27 European American and 23 African American) that passed quality control metrics. Overall, we observed similar characteristics between the European and African American groups, with the exception of BMI and hypertension. African Americans in this cohort were more hypertensive despite having lower BMI. Characteristics of the study cohort are provided in Table 1.

**Table 1:** Population demographics and characteristics.

	Overall (N = 50)	European American (N = 27)	African American (N = 23)	p-value*
**Age (years)**	27.3 ± 3.7	27 ± 3.0	28 ± 4.3	0.329
**BMI**	25.1 ± 4.2	26.2 ± 3.9	23.7 ± 4.3	0.044
**Hypertensive, n (%)**	9 (17.3%)	2 (6.9%)	7 (30.4%)	0.035
**HDL (mg/dL)**	52.8 ± 21.9	47.6 ± 17.5	59.5 ± 25.7	0.067
**Non-HDL (mg/dL)**	147.9 ± 88.3	169.4 ± 88.6	122.8 ± 82.9	0.061
**Thiocyanate (uMol/L)**	85.9 ± 40.9	88.2 ± 44.9	83.3 ± 36.4	0.676
**Glycohemoglobin (%)**	6.5 ± 0.8	6.6 ± 0.8	6.5 ± 0.7	0.610

Means and standard deviations are reported.

*p-values were calculated using two-sample t-test or Chi-square test to evaluate differences between racial groups.

**Fig 2:**
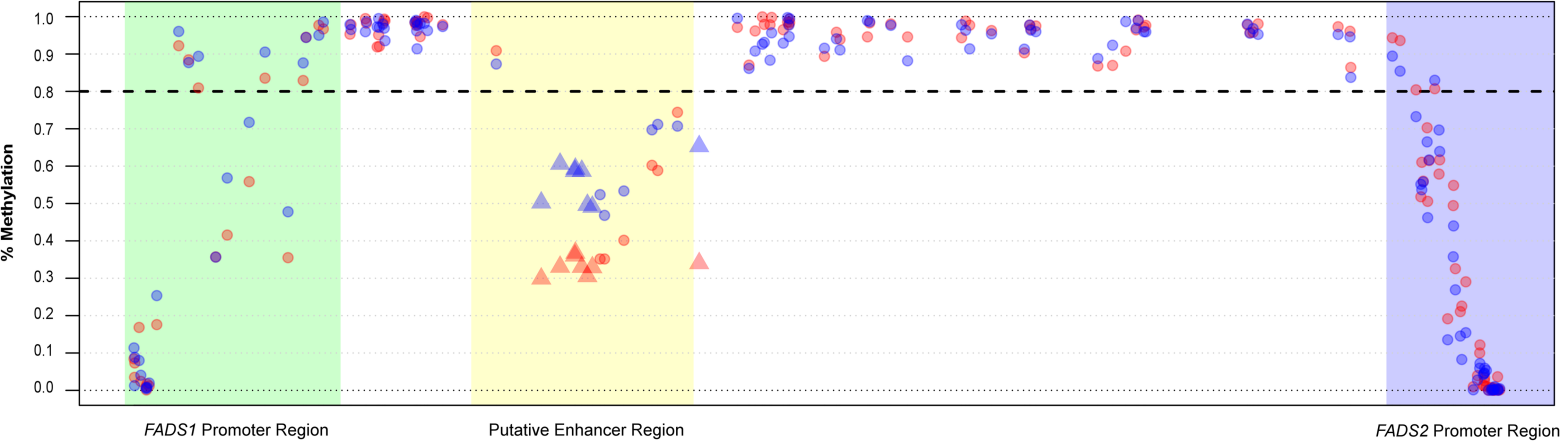
Illustration of DNA methylation landscape across the sequenced region for European and African Americans. Average DNA methylation levels for European Americans are denoted by blue circles and average DNA methylation for African Americans are shown as red circles. Highlighted regions correspond to the *FADS1* promoter (green), putative enhancer (yellow) and *FADS2* promoter (blue) regions. Dashed line represents 80% methylation. Triangle shaped points in the putative enhancer region are CpG sites that displayed significant ASM with rs174537 in the meta-analysis.

Examining the average methylation status across the region revealed that variance in methylation was greatest within the three key regulatory regions (Fig 2). CpG sites outside of these three regions displayed high levels of methylation (>80% methylation, on average). Further investigation demonstrated substantial variability in the methylation status of CpG sites located in the putative enhancer region (Fig 2).

**Fig 3:**
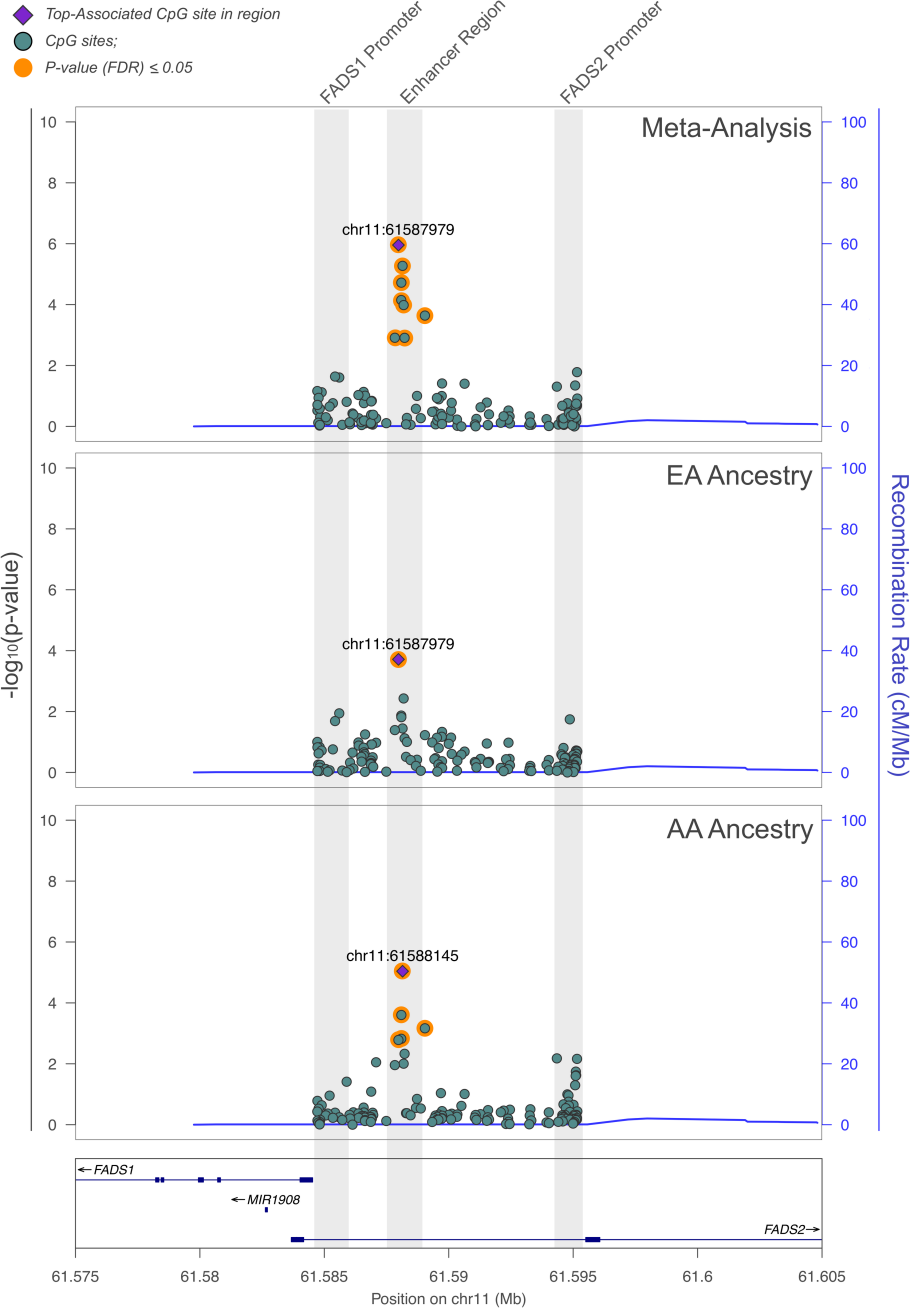
Manhattan plot for association of rs174537 with CpG sites from the sequenced region between *FADS1* and *FADS2*, showing the meta-analysis, and stratified by racial group. European Americans are denoted by EA and African Americans are denoted by AA. CpG sites highlighted in orange displayed significant ASM associations (FDR P-value <0.05), based on the genetic trend test that was adjusted for age. The blue line represents the recombination rate.

To assess the association of rs174537 with the methylation status of the 136 CpG sites, a linear regression was computed, adjusting for age. In the overall, race-combined meta-analysis, the greatest ASM with rs174537 occurred at the CpG site located at chr11:61587979 (i.e., cg27386326; Fig 3). This was the same site that demonstrated the greatest ASM (p = 2.69×10^−29^) with rs174537 in our previous methylation GWAS study. The meta-analysis across the region also revealed an additional seven significantly associated (FDR <0.05) CpG sites (a total of eight), which were localized within the putative enhancer region (61587835, 61587979, 61588092, 61588096, 61588145, 61588226, and 61589043) (Fig 3, Table 1, FDR p-value<0.05). Interestingly, ASM associations stratified by race identified some CpG sites in the *FADS1* and *FADS2* promoter regions (Table 2). While these sites did not meet the FDR level of significance, they show promise for further investigation in larger cohorts. The complete ASM results for all 136 CpG sites with their Cg IDs have been provided in S1 Table (S1 Table).

**Table 2:** Strongest associations between SNP rs174537 (coded for dominant genetic model, relative to T-allele) and CpG sites within the *FADS1* and *FADS2* sequenced region. (A) Meta-analysis including both races, (B) Stratified analysis for European Americans only, and (C) Stratified analysis for African Americans only.

**A.**						
**Meta-Analysis (N = 50)**						
**CpG Site**	**Region**			**P-value**		**FDR**
chr11:61587979*	Putative Enhancer Region			1.11E-06		1.51E-04
chr11:61588145	Putative Enhancer Region			5.34E-06		3.63E-04
chr11:61588096	Putative Enhancer Region			1.90E-05		8.62E-04
chr11:61588092	Putative Enhancer Region			7.09E-05		2.41E-03
chr11:61588188	Putative Enhancer Region			1.04E-04		2.82E-03
chr11:61589043	Putative Enhancer Region			2.29E-04		5.19E-03
chr11:61587835	Putative Enhancer Region			1.23E-03		2.10E-02
chr11:61588226	Putative Enhancer Region			1.23E-03		2.10E-02
**B.**						
**European Americans (N = 27)**						
**CpG Site**	**Region**	**Estimate**	**Std Err**	**T-value**	**P-value**	**FDR**
chr11:61587979*	Putative Enhancer Region	0.41327	0.09255	4.47	1.94E-04	2.63E-02
chr11:61588188	Putative Enhancer Region	0.24895	0.07745	3.21	3.71E-03	2.52E-01
chr11:61585601	*FADS1* Promoter Region	0.31678	0.11525	2.75	1.14E-02	3.98E-01
chr11:61588092	Putative Enhancer Region	0.26866	0.10043	2.67	1.35E-02	3.98E-01
chr11:61588096	Putative Enhancer Region	0.27249	0.10399	2.62	1.53E-02	3.98E-01
chr11:61594865	*FADS2* Promoter Region	0.32601	0.11745	2.78	1.80E-02	3.98E-01
chr11:61585433	*FADS1* Promoter Region	0.28853	0.11556	2.50	2.05E-02	3.98E-01
**C.**						
**African Americans (N = 23)**						
**CpG Site**	**Region**	**Estimate**	**Std Err**	**T-value**	**P-value**	**FDR**
chr11:61588145	Putative Enhancer Region	0.41103	0.06973	5.89	9.13E-06	1.24E-03
chr11:61588096	Putative Enhancer Region	0.38883	0.0875	4.44	2.49E-04	1.70E-02
chr11:61589043	Putative Enhancer Region	0.47099	0.11505	4.09	6.81E-04	3.09E-02
chr11:61588092	Putative Enhancer Region	0.33793	0.09198	3.67	1.51E-03	4.48E-02
chr11:61587979*	Putative Enhancer Region	0.38706	0.10563	3.66	1.65E-03	4.48E-02
chr11:61588226	Putative Enhancer Region	0.27146	0.08531	3.18	4.69E-03	1.06E-01
chr11:61594345	*FADS2* Promoter Region	-0.14713	0.04828	-3.05	6.63E-03	1.17E-01

*CgID is only available for CpG site chr11:61587979 (i.e. cg27386326)

In many cases, we observed methylation levels of adjacent CpG sites to be correlated with one another. For example, adjacent CpG sites to chr11:61587979 (i.e. cg27386326), namely 61588092 and 61588096, were highly correlated (r = 0.94), suggesting that CpG sites within close proximity to one another can experience similar levels of methylation. Similarly, three CpG sites within the *FADS2* promoter region (i.e. 61594865, 61594876, 61594907, r = 0.86) were strongly correlated with each other. While CpG site 61594865 was the only one that displayed trends towards a strong ASM association with rs175537 in the European American population, it is possible that these adjacent CpG sites could be just as influential in larger studies.

**Fig 4:**
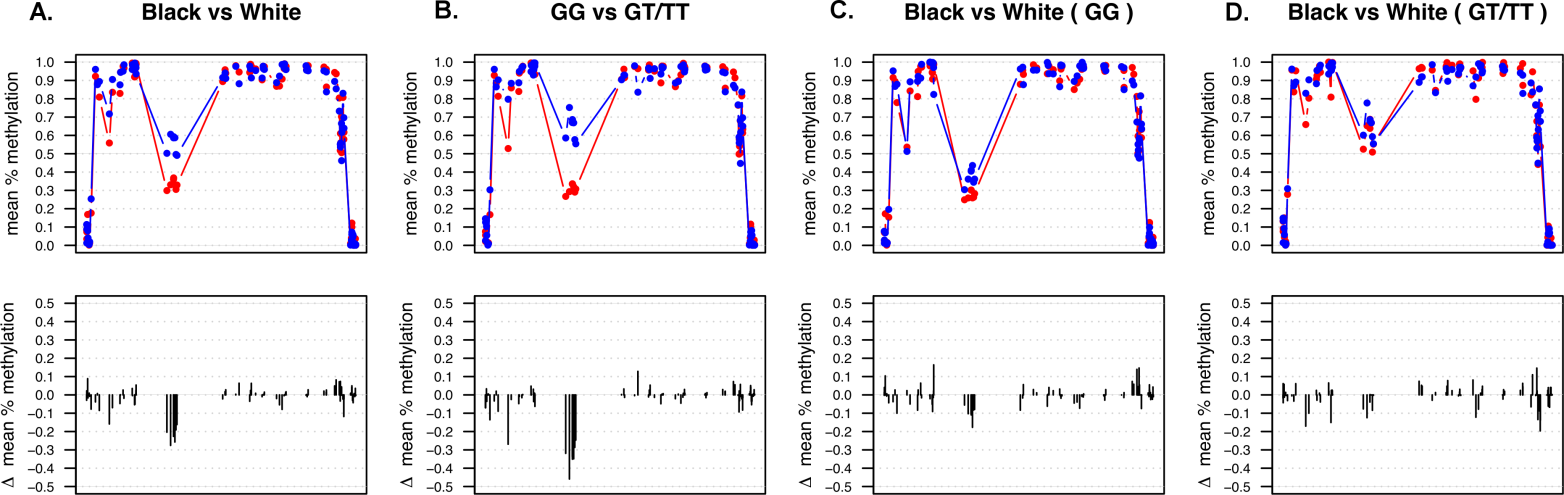
Mean proportion of DNA methylation across the sequenced region is dependent on genotype at rs174537. (A) Racial differences observed in the enhancer region (African American–red; European American–blue). (B) Genotype at rs174537 affects methylation status in the enhancer region (GG–red; GT/TT–blue). (C) No statistically significant differences between African American and European American both with GG genotype. (D) No statistically significant differences between African American and European American both with GT/TT genotype.

While the data from Fig 2 appears to indicate that there are differences in methylation status by race within these three key regulatory regions, this is in fact explained by differences in genotype, with the allele frequencies of rs174537 being different by race. Analysis stratified by race (Figs 2 & 4) reveal that the European Americans displayed lower levels of methylation, on average, at CpG sites within the *FADS2* promoter region compared to African American subjects. In contrast, African Americans expressed lower methylation at CpG sites 61587979 (i.e. cg27386326), 61588145 and 61589043 within the enhancer region. However, the racial differences in the methylation status of CpG sites within the enhancer region indicated in Fig 2 were observed to be strongly dependent on genotype at rs174537 (Fig 4). When stratified by race, CpG site chr11:61587979 (i.e. cg27386326) continued to display the strongest ASM association with rs174537 in European Americans. However, the strongest ASM was observed at CpG 61588145 in African Americans; this site is only 166 bp away from 61587979 (i.e. cg27386326) and also located in the putative enhancer region. Table 2 reports the CpG sites with the strongest ASM associations for European and African Ancestry populations and the overall meta-analysis for this cohort.

## Discussion

Omega-6 and omega-3 LC-PUFAs such as arachidonic acid (ARA) and docosahexaenoic acid (DHA) have long been recognized to have vital structural roles in cellular membranes, brain development and function, and inflammation [39–41]. Given this strong relationship between LC-PUFAs and human physiology and recent evidence demonstrating the significant impact of genomic variants, including rs174537, on LC-PUFA levels and related phenotypes, we conducted a more in-depth investigation of the methylation status of 136 CpG sites between *FADS1* and *FADS2* genes. Our goals were to 1) characterize the DNA methylation landscape within this potentially important regulatory area; 2) identify new ASM associations with genotype at rs174537; and 3) better understand the molecular mechanism by which genetic and epigenetic variations with the *FADS* cluster may influence LC-PUFA biosynthesis in human tissues.

Seven new CpG sites were associated within the sequenced region, in addition to the previously identified CpG site 61587979 (i.e. cg27386326), totaling eight CpG sites exhibiting significant ASM associations with rs174537. CpG sites that displayed the greatest ASM association with rs174537 were localized within the putative enhancer region. These associations were replicated within each ethnic group and strengthened by the meta-analysis. Notably, there were additional signals of suggestive significance that may be enhanced with larger sample sizes. For example, European Americans showed suggestive associations at both the *FADS1* (p = 0.014) and *FADS2* (p = 0.018) promoter regions; and African Americans showed a suggestive *FADS2* promoter signal (p = 0.006) which was supported by CpG sites in close-proximity (Fig 3).

The DNA methylation landscape of the sequenced region revealed areas of hyper- and hypo-methylation, with the three key regulatory regions (the *FADS1* promoter, the putative enhancer, and the *FADS2* promoter regions) displaying the greatest variation in methylation levels. In general, we observed lower average levels of methylation (<25%) in the two promoter regions. Provided that increased DNA methylation at promoter regions is generally associated with lower gene expression levels, it is likely that *FADS1* and *FADS2* gene expression levels were not greatly suppressed in this cohort. Ultimately, this needs to be experimentally tested. Average methylation levels in the putative enhancer region were generally higher than the promoter regions (~50%), but displayed a great deal of variability between subjects, ethnicity, and genotype, with genotype being the most influential factor.

Individuals homozygous for the major allele (i.e. GG genotype at rs17547), regardless of race, displayed the lowest levels of methylation within the putative enhancer region (Fig 4). Given that the GG genotype is associated with increased metabolic conversion capacities of dietary PUFAs to LC-PUFAs, it is possible that their low levels of methylation does not suppress *FADS1* and *FADS2* gene expression. Alternatively, individuals with GT and TT genotypes who have slower metabolic conversion capacities, with TT subjects exhibiting deficiencies in LC-PUFAs, could be experiencing reduced *FADS1* and *FADS2* expression levels due to their increased DNA methylation. This suggests that reducing methylation levels in this regulatory region for individuals having GT and/or TT genotypes could potentially improve their endogenous synthesis of LC-PUFAs.

It is clear that there is some relationship between the rs174537 genotype and methylation in the sequenced region, and that this relationship has the potential to strongly impact LC-PUFA biosynthesis. It is unlikely that the rs174537 genotype affects the methylation status directly within these regulatory regions, since it resides over 30kb from the associated CpG sites.rs174537 resides in a large LD block, so other SNPs within this block and closer to the regulatory region would show similar relationships. Further studies are necessary to better understand the three-dimensional architecture of the *FADS* cluster and to determine if these genetic and epigenetic variants interact in that context.

Epigenetic regulation has emerged as an important research focus, with environmental and particularly dietary exposure being key factors that influence PUFA metabolism and *FADS* gene expression levels. For example, Hoile et al. recently reported significant changes in DNA methylation of CpG sites within the *FADS* region in response to changing the fatty acid composition of the human diet [42]. Specifically, they discovered three CpG sites in the *FADS2* promoter region to be most influenced by changes in diet. Interestingly, the single CpG site we identified in the *FADS2* promoter within the European American cohort in this study (i.e. 61594865) is also one that Hoile et al. found to be impacted by diet. Together, these data imply that, in addition to being highly associated with rs174537, these CpG sites could play a pivotal role in sensing dietary, circulating, and tissue PUFA levels, and potentially regulate *FADS* gene transcription.

Genomic variants near and within the *FADS* cluster have been shown to influence downstream processes from the PUFA biosynthesis pathway, including inflammation, cardiovascular disease, cancer, and other chronic diseases. For example, maternal fat intake in rats has been shown to alter the epigenetic regulation of *FADS2* in offspring liver. Additionally, the methylation status of CpG sites in two critical regulatory regions of the *FADS* cluster have been shown to be associated with blood and tissue PUFA levels as well as an important phenotype (e.g. immediate and intermediate memory) in toddlers [43, 44]. It is clear that further investigation is needed to tease out the importance of the methylation status of CpG sites in this important *FADS* regulatory region.

This study, along with previous work from our lab, has demonstrated the significant racial differences in the genetic and epigenetic status of variants in the *FADS* cluster as well as LC-PUFA biosynthesis with African Ancestry populations having higher frequencies of variants associated with elevated LC-PUFA levels. We postulate that these genomic variants, along with high omega-6 PUFA exposure in the modern Western diet, have the capacity to create destructive genetic/epigenetic-dietary PUFA interactions. This, in turn, could have important implications on downstream inflammatory processes and disease states, including hypertension, diabetes, heart disease, and cancer [35, 36, 45, 46]. Taken together, all of these studies suggest that a “one size fits all” dietary PUFA recommendation may not be appropriate. Further work is clearly needed to investigate the impact of these genomic variants on these proposed disease states in ethnically diverse populations.

While the DNA methylation landscape of this region has been well characterized and strong ASM associations with rs174537 have been identified in this limited study cohort, the methylation status of additional CpG sites may strongly influence circulating and tissue PUFA levels. A limitation of this work is that while it provided important new data regarding the methylation status of key CpG sites within the 12kb region of the *FADS* cluster, there may be numerous other regulatory mechanisms that impact LC-PUFA biosynthesis. Another limitation is due to the relatively small number of liver samples; it was not possible to power the study to evaluate the impact of the methylation of these CpG sites on PUFA levels. However, this work points out specific CpG sites where the methylation status is likely to affect LC-PUFA biosynthesis, and thus provides important knowledge to the field regarding which CpG sites to examine in future studies. Furthermore, it is encouraging that the CpG site (chr11:61594865) we identified in the *FADS2* promoter region using ASM, for the European American population, was also identified by Hoile et al. [42] from whole blood samples, suggesting that there is overlap between DNA methylation in liver tissue and circulating blood cells for these specific CpG sites.

## Conclusions

In conclusion, this in-depth characterization of the region between *FADS1* and *FADS2* genes revealed eight key CpG sites that were associated with genotype at rs174537. These data validate our previous observation of ASM between rs174537 and the methylation status of cg27386326 located in the putative enhancer region. Additionally, seven new key CpG sites were identified, based on FDR significance. Taken together, these data show a significant association between the genomic region tagged by rs174537 and DNA methylation in the putative enhancer region. Further investigation is needed to determine if altering the DNA methylation landscape at this region can influence *FADS1* and *FADS2* gene expression levels, ultimately impacting metabolic conversion capacities and the overall synthesis of LC-PUFAs in humans. In addition, it is unknown if alterations in the DNA methylation landscape will preferentially benefit individuals with certain genotypes (e.g. GG vs. TT). Needless to say, this study highlights the importance of genetic and epigenetic factors that may strongly impact the capacity of tissues such as the liver to synthesize LC-PUFAs.

## Supporting information

S1 Table ASM results stratified by race and meta-analyses for all 136 CpG sites. The estimates, standard errors, raw p-values and FDR p-values are reported for each CpG site. The *FADS1* promoter (green), putative enhancer (yellow) and *FADS2* promoter (blue) regions are highlighted, using the same color scheme as in the manuscript. The name of each CpG is based on its genomic position on chromosome 11 (0-based format), based on genome build GRCh37/hg19; cg IDs are provided when available.
(XLSX)

S1 File DNA methylation data from study cohort. All relevant data pertaining to this study, including demographics, clinical variables, and CpG sites, have been provided in the form of a supplemental excel file. All data is de-identified and a data dictionary has been provided within the file.
(XLSX)

## References

[1] Bazinet RP, Laye S. Polyunsaturated fatty acids and their metabolites in brain function and disease. Nat Rev Neurosci. 2014;15(12):771–85. doi: 10.1038/nrn3820 . 25387473 10.1038/nrn3820

[2] Berquin IM, Edwards IJ, Kridel SJ, Chen YQ. Polyunsaturated fatty acid metabolism in prostate cancer. Cancer Metastasis Rev. 2011;30(3–4):295–309. doi: 10.1007/s10555-011-9299-7 ; PubMed Central PMCID: PMCPMC3865857. 22015690 10.1007/s10555-011-9299-7 PMC3865857

[3] Simopoulos AP. The importance of the omega-6/omega-3 fatty acid ratio in cardiovascular disease and other chronic diseases. Exp Biol Med (Maywood). 2008;233(6):674–88. doi: 10.3181/0711-MR-311 . 18408140 10.3181/0711-MR-311

[4] Mozaffarian D, Wu JH. Omega-3 fatty acids and cardiovascular disease: effects on risk factors, molecular pathways, and clinical events. J Am Coll Cardiol. 2011;58(20):2047–67. doi: 10.1016/j.jacc.2011.06.063 . 22051327 10.1016/j.jacc.2011.06.063

[5] Malerba G, Schaeffer L, Xumerle L, Klopp N, Trabetti E, Biscuola M, et al SNPs of the FADS gene cluster are associated with polyunsaturated fatty acids in a cohort of patients with cardiovascular disease. Lipids. 2008;43(4):289–99. doi: 10.1007/s11745-008-3158-5 . 18320251 10.1007/s11745-008-3158-5

[6] Martinelli N, Girelli D, Malerba G, Guarini P, Illig T, Trabetti E, et al FADS genotypes and desaturase activity estimated by the ratio of arachidonic acid to linoleic acid are associated with inflammation and coronary artery disease. Am J Clin Nutr. 2008;88(4):941–9. . 18842780 10.1093/ajcn/88.4.941

[7] Rzehak P, Heinrich J, Klopp N, Schaeffer L, Hoff S, Wolfram G, et al Evidence for an association between genetic variants of the fatty acid desaturase 1 fatty acid desaturase 2 (FADS1 FADS2) gene cluster and the fatty acid composition of erythrocyte membranes. British Journal of Nutrition. 2009;101(1):20–6. doi: 10.1017/S0007114508992564 18479586 10.1017/S0007114508992564

[8] Schaeffer L, Gohlke H, Muller M, Heid IM, Palmer LJ, Kompauer I, et al Common genetic variants of the FADS1 FADS2 gene cluster and their reconstructed haplotypes are associated with the fatty acid composition in phospholipids. Human Molecular Genetics. 2006;15(11):1745–56. doi: 10.1093/hmg/ddl117 16670158 10.1093/hmg/ddl117

[9] Mathias RA, Sergeant S, Ruczinski I, Torgerson DG, Hugenschmidt CE, Kubala M, et al The impact of FADS genetic variants on omega6 polyunsaturated fatty acid metabolism in African Americans. BMC Genet. 2011;12:50 doi: 10.1186/1471-2156-12-50 ; PubMed Central PMCID: PMCPMC3118962. 21599946 10.1186/1471-2156-12-50 PMC3118962

[10] Mathias RA, Vergara C, Gao L, Rafaels N, Hand T, Campbell M, et al FADS genetic variants and omega-6 polyunsaturated fatty acid metabolism in a homogeneous island population. J Lipid Res. 2010;51(9):2766–74. doi: 10.1194/jlr.M008359 ; PubMed Central PMCID: PMCPMC2918459. 20562440 10.1194/jlr.M008359 PMC2918459

[11] Sergeant S, Hugenschmidt CE, Rudock ME, Ziegler JT, Ivester P, Ainsworth HC, et al Differences in arachidonic acid levels and fatty acid desaturase (FADS) gene variants in African Americans and European Americans with diabetes or the metabolic syndrome. Br J Nutr. 2012;107(4):547–55. doi: 10.1017/S0007114511003230 ; PubMed Central PMCID: PMCPMC3494092. 21733300 10.1017/S0007114511003230 PMC3494092

[12] Xie L, Innis SM. Genetic variants of the FADS1 FADS2 gene cluster are associated with altered (n-6) and (n-3) essential fatty acids in plasma and erythrocyte phospholipids in women during pregnancy and in breast milk during lactation. J Nutr. 2008;138(11):2222–8. doi: 10.3945/jn.108.096156 . 18936223 10.3945/jn.108.096156

[13] Xie L, Innis SM. Association of Fatty Acid Desaturase Gene Polymorphisms with Blood Lipid Essential Fatty Acids and Perinatal Depression among Canadian Women: A Pilot Study. Journal of Nutrigenetics and Nutrigenomics. 2009;2(4–5):243–50. doi: 10.1159/000255636 20395685 10.1159/000255636

[14] Porenta SR, Ko YA, Gruber SB, Mukherjee B, Baylin A, Ren J, et al Interaction of fatty acid genotype and diet on changes in colonic fatty acids in a Mediterranean diet intervention study. Cancer Prev Res (Phila). 2013;6(11):1212–21. doi: 10.1158/1940-6207.CAPR-13-0131 ; PubMed Central PMCID: PMCPMC3840911. 24022589 10.1158/1940-6207.CAPR-13-0131 PMC3840911

[15] Hong SH, Kwak JH, Paik JK, Chae JS, Lee JH. Association of polymorphisms in FADS gene with age-related changes in serum phospholipid polyunsaturated fatty acids and oxidative stress markers in middle-aged nonobese men. Clin Interv Aging. 2013;8:585–96. doi: 10.2147/CIA.S42096 ; PubMed Central PMCID: PMCPMC3693593. 23818766 10.2147/CIA.S42096 PMC3693593

[16] Harslof LB, Larsen LH, Ritz C, Hellgren LI, Michaelsen KF, Vogel U, et al FADS genotype and diet are important determinants of DHA status: a cross-sectional study in Danish infants. Am J Clin Nutr. 2013;97(6):1403–10. doi: 10.3945/ajcn.113.058685 . 23636240 10.3945/ajcn.113.058685

[17] Li SW, Lin K, Ma P, Zhang ZL, Zhou YD, Lu SY, et al FADS gene polymorphisms confer the risk of coronary artery disease in a Chinese Han population through the altered desaturase activities: based on high-resolution melting analysis. PLoS One. 2013;8(1):e55869 doi: 10.1371/journal.pone.0055869 ; PubMed Central PMCID: PMCPMC3561316. 23383292 10.1371/journal.pone.0055869 PMC3561316

[18] Morales E, Bustamante M, Gonzalez JR, Guxens M, Torrent M, Mendez M, et al Genetic variants of the FADS gene cluster and ELOVL gene family, colostrums LC-PUFA levels, breastfeeding, and child cognition. PLoS One. 2011;6(2):e17181 doi: 10.1371/journal.pone.0017181 ; PubMed Central PMCID: PMCPMC3044172. 21383846 10.1371/journal.pone.0017181 PMC3044172

[19] Gillingham LG, Harding SV, Rideout TC, Yurkova N, Cunnane SC, Eck PK, et al Dietary oils and FADS1-FADS2 genetic variants modulate [13C]alpha-linolenic acid metabolism and plasma fatty acid composition. Am J Clin Nutr. 2013;97(1):195–207. doi: 10.3945/ajcn.112.043117 . 23221573 10.3945/ajcn.112.043117

[20] Freemantle E, Lalovic A, Mechawar N, Turecki G. Age and haplotype variations within FADS1 interact and associate with alterations in fatty acid composition in human male cortical brain tissue. PLoS One. 2012;7(8):e42696 doi: 10.1371/journal.pone.0042696 ; PubMed Central PMCID: PMCPMC3416866. 22900039 10.1371/journal.pone.0042696 PMC3416866

[21] Lattka E, Koletzko B, Zeilinger S, Hibbeln JR, Klopp N, Ring SM, et al Umbilical cord PUFA are determined by maternal and child fatty acid desaturase (FADS) genetic variants in the Avon Longitudinal Study of Parents and Children (ALSPAC). Br J Nutr. 2013;109(7):1196–210. doi: 10.1017/S0007114512003108 ; PubMed Central PMCID: PMCPMC3600399. 22877655 10.1017/S0007114512003108 PMC3600399

[22] Lattka E, Rzehak P, Szabo E, Jakobik V, Weck M, Weyermann M, et al Genetic variants in the FADS gene cluster are associated with arachidonic acid concentrations of human breast milk at 1.5 and 6 mo postpartum and influence the course of milk dodecanoic, tetracosenoic, and trans-9-octadecenoic acid concentrations over the duration of lactation. Am J Clin Nutr. 2011;93(2):382–91. doi: 10.3945/ajcn.110.004515 . 21147856 10.3945/ajcn.110.004515

[23] Steer CD, Hibbeln JR, Golding J, Smith GD. Polyunsaturated fatty acid levels in blood during pregnancy, at birth and at 7 years: their associations with two common FADS2 polymorphisms. Human Molecular Genetics. 2012;21(7):1504–12. doi: 10.1093/hmg/ddr588 22194195 10.1093/hmg/ddr588 PMC3465695

[24] Koletzko B, Lattka E, Zeilinger S, Illig T, Steer C. Genetic variants of the fatty acid desaturase gene cluster predict amounts of red blood cell docosahexaenoic and other polyunsaturated fatty acids in pregnant women: findings from the Avon Longitudinal Study of Parents and Children. Am J Clin Nutr. 2011;93(1):211–9. doi: 10.3945/ajcn.110.006189 . 21106917 10.3945/ajcn.110.006189

[25] Kwak JH, Paik JK, Kim OY, Jang Y, Lee SH, Ordovas JM, et al FADS gene polymorphisms in Koreans: association with omega6 polyunsaturated fatty acids in serum phospholipids, lipid peroxides, and coronary artery disease. Atherosclerosis. 2011;214(1):94–100. doi: 10.1016/j.atherosclerosis.2010.10.004 . 21040914 10.1016/j.atherosclerosis.2010.10.004

[26] Rzehak P, Thijs C, Standl M, Mommers M, Glaser C, Jansen E, et al Variants of the FADS1 FADS2 Gene Cluster, Blood Levels of Polyunsaturated Fatty Acids and Eczema in Children within the First 2 Years of Life. Plos One. 2010;5(10):e13261 ARTN e13261 doi: 10.1371/journal.pone.0013261 20948998 10.1371/journal.pone.0013261 PMC2952585

[27] Bokor S, Dumont J, Spinneker A, Gonzalez-Gross M, Nova E, Widhalm K, et al Single nucleotide polymorphisms in the FADS gene cluster are associated with delta-5 and delta-6 desaturase activities estimated by serum fatty acid ratios. J Lipid Res. 2010;51(8):2325–33. doi: 10.1194/jlr.M006205 ; PubMed Central PMCID: PMCPMC2903808. 20427696 10.1194/jlr.M006205 PMC2903808

[28] Glaser C, Heinrich J, Koletzko B. Role of FADS1 and FADS2 polymorphisms in polyunsaturated fatty acid metabolism. Metabolism: clinical and experimental. 2010;59(7):993–9. Epub 2010/01/05. doi: 10.1016/j.metabol.2009.10.022 . 20045144 10.1016/j.metabol.2009.10.022

[29] Tanaka T, Shen J, Abecasis GR, Kisialiou A, Ordovas JM, Guralnik JM, et al Genome-wide association study of plasma polyunsaturated fatty acids in the InCHIANTI Study. PLoS Genet. 2009;5(1):e1000338 doi: 10.1371/journal.pgen.1000338 ; PubMed Central PMCID: PMCPMC2613033. 19148276 10.1371/journal.pgen.1000338 PMC2613033

[30] Kathiresan S, Melander O, Guiducci C, Surti A, Burtt NP, Rieder MJ, et al Six new loci associated with blood low-density lipoprotein cholesterol, high-density lipoprotein cholesterol or triglycerides in humans. Nat Genet. 2008;40(2):189–97. doi: 10.1038/ng.75 ; PubMed Central PMCID: PMCPMC2682493. 18193044 10.1038/ng.75 PMC2682493

[31] Willer CJ, Sanna S, Jackson AU, Scuteri A, Bonnycastle LL, Clarke R, et al Newly identified loci that influence lipid concentrations and risk of coronary artery disease. Nature Genetics. 2008;40(2):161–9. doi: 10.1038/ng.76 18193043 10.1038/ng.76 PMC5206900

[32] Lu Y, Vaarhorst A, Merry AH, Dolle ME, Hovenier R, Imholz S, et al Markers of endogenous desaturase activity and risk of coronary heart disease in the CAREMA cohort study. PLoS One. 2012;7(7):e41681 doi: 10.1371/journal.pone.0041681 ; PubMed Central PMCID: PMCPMC3402436. 22911844 10.1371/journal.pone.0041681 PMC3402436

[33] Brookes KJ, Chen W, Xu X, Taylor E, Asherson P. Association of fatty acid desaturase genes with attention-deficit/hyperactivity disorder. Biological psychiatry. 2006;60(10):1053–61. Epub 2006/08/09. doi: 10.1016/j.biopsych.2006.04.025 . 16893529 10.1016/j.biopsych.2006.04.025

[34] Howard TD, Mathias RA, Seeds MC, Herrington DM, Hixson JE, Shimmin LC, et al DNA methylation in an enhancer region of the FADS cluster is associated with FADS activity in human liver. PLoS One. 2014;9(5):e97510 doi: 10.1371/journal.pone.0097510 ; PubMed Central PMCID: PMCPMC4026313. 24842322 10.1371/journal.pone.0097510 PMC4026313

[35] Chilton FH, Murphy RC, Wilson BA, Sergeant S, Ainsworth H, Seeds MC, et al Diet-gene interactions and PUFA metabolism: a potential contributor to health disparities and human diseases. Nutrients. 2014;6(5):1993–2022. doi: 10.3390/nu6051993 ; PubMed Central PMCID: PMCPMC4042578. 24853887 10.3390/nu6051993 PMC4042578

[36] Wilson BA, Sergeant S, Ainsworth H, Mathias R, Chilton FH. Racial Differences in Plasma Omega-3 Long Chain Fatty Acid Levels in a Cohort of African Americans and European Americans with Diabetes and Metabolic Syndrome. Faseb Journal. 2012;26. WOS:000310711305363.

[37] Strong JP, Malcom GT, Oalmann MC, Wissler RW. The PDAY Study: natural history, risk factors, and pathobiology. Pathobiological Determinants of Atherosclerosis in Youth. Ann N Y Acad Sci. 1997;811:226–35; discussion 35–7. . 9186600 10.1111/j.1749-6632.1997.tb52004.x

[38] Willer CJ, Li Y, Abecasis GR. METAL: fast and efficient meta-analysis of genomewide association scans. Bioinformatics. 2010;26(17):2190–1. doi: 10.1093/bioinformatics/btq340 ; PubMed Central PMCID: PMCPMC2922887. 20616382 10.1093/bioinformatics/btq340 PMC2922887

[39] Hester AG, Murphy RC, Uhlson CJ, Ivester P, Lee TC, Sergeant S, et al Relationship between a common variant in the fatty acid desaturase (FADS) cluster and eicosanoid generation in humans. J Biol Chem. 2014;289(32):22482–9. Epub 2014/06/26. doi: 10.1074/jbc.M114.579557 ; PubMed Central PMCID: PMCPMC4139254. 24962583 10.1074/jbc.M114.579557 PMC4139254

[40] Liu TF, Brown CM, El Gazzar M, McPhail L, Millet P, Rao A, et al Fueling the flame: bioenergy couples metabolism and inflammation. Journal of leukocyte biology. 2012;92(3):499–507. doi: 10.1189/jlb.0212078 ; PubMed Central PMCID: PMCPMC3427613. 22571857 10.1189/jlb.0212078 PMC3427613

[41] Weaver KL, Ivester P, Seeds M, Case LD, Arm JP, Chilton FH. Effect of dietary fatty acids on inflammatory gene expression in healthy humans. J Biol Chem. 2009;284(23):15400–7. Epub 2009/04/11. doi: 10.1074/jbc.M109.004861 ; PubMed Central PMCID: PMCPMC2708836. 19359242 10.1074/jbc.M109.004861 PMC2708836

[42] Hoile SP, Clarke-Harris R, Huang RC, Calder PC, Mori TA, Beilin LJ, et al Supplementation with N-3 long-chain polyunsaturated fatty acids or olive oil in men and women with renal disease induces differential changes in the DNA methylation of FADS2 and ELOVL5 in peripheral blood mononuclear cells. PLoS One. 2014;9(10):e109896 doi: 10.1371/journal.pone.0109896 ; PubMed Central PMCID: PMCPMC4201459. 25329159 10.1371/journal.pone.0109896 PMC4201459

[43] Cheatham CL, Lupu DS, Niculescu MD. Genetic and epigenetic transgenerational implications related to omega-3 fatty acids. Part II: maternal FADS2 rs174575 genotype and DNA methylation predict toddler cognitive performance. Nutr Res. 2015;35(11):948–55. doi: 10.1016/j.nutres.2015.09.005 . 26455892 10.1016/j.nutres.2015.09.005

[44] Lupu DS, Cheatham CL, Corbin KD, Niculescu MD. Genetic and epigenetic transgenerational implications related to omega-3 fatty acids. Part I: maternal FADS2 genotype and DNA methylation correlate with polyunsaturated fatty acid status in toddlers: an exploratory analysis. Nutr Res. 2015;35(11):939–47. doi: 10.1016/j.nutres.2015.09.004 . 26439440 10.1016/j.nutres.2015.09.004

[45] Cui T, Hester AG, Seeds MC, Rahbar E, Howard TD, Sergeant S, et al Impact of Genetic and Epigenetic Variations Within the FADS Cluster on the Composition and Metabolism of Polyunsaturated Fatty Acids in Prostate Cancer. Prostate. 2016;76(13):1182–91. doi: 10.1002/pros.23205 . 27197070 10.1002/pros.23205 PMC6680327

[46] Mathias RA, Pani V, Chilton FH. Genetic Variants in the FADS Gene: Implications for Dietary Recommendations for Fatty Acid Intake. Current nutrition reports. 2014;3(2):139–48. Epub 2014/07/01. doi: 10.1007/s13668-014-0079-1 ; PubMed Central PMCID: PMCPMC4070521. 24977108 10.1007/s13668-014-0079-1 PMC4070521

